# Novel Vaccine Adjuvants as Key Tools for Improving Pandemic Preparedness

**DOI:** 10.3390/bioengineering8110155

**Published:** 2021-10-24

**Authors:** Brett H. Pogostin, Kevin J. McHugh

**Affiliations:** Department of Bioengineering, Rice University, Houston, TX 77030, USA; bp22@rice.edu

**Keywords:** adjuvants, vaccines, pandemic, infectious disease, platform technology, humoral immunity, cellular immunity, antigen, innate immunity, global health

## Abstract

Future infectious disease outbreaks are inevitable; therefore, it is critical that we maximize our readiness for these events by preparing effective public health policies and healthcare innovations. Although we do not know the nature of future pathogens, antigen-agnostic platforms have the potential to be broadly useful in the rapid response to an emerging infection—particularly in the case of vaccines. During the current COVID-19 pandemic, recent advances in mRNA engineering have proven paramount in the rapid design and production of effective vaccines. Comparatively, however, the development of new adjuvants capable of enhancing vaccine efficacy has been lagging. Despite massive improvements in our understanding of immunology, fewer than ten adjuvants have been approved for human use in the century since the discovery of the first adjuvant. Modern adjuvants can improve vaccines against future pathogens by reducing cost, improving antigen immunogenicity, and increasing antigen stability. In this perspective, we survey the current state of adjuvant use, highlight potentially impactful preclinical adjuvants, and propose new measures to accelerate adjuvant safety testing and technology sharing to enable the use of “off-the-shelf” adjuvant platforms for rapid vaccine testing and deployment in the face of future pandemics.

## 1. Introduction

In the modern hyperconnected world, deadly global pandemics are more likely than ever. Even before the coronavirus disease 2019 (COVID-19) pandemic, 3 of the top 10 leading causes of death worldwide were infectious diseases [1]. The burden of communicable disease, however, is not distributed evenly. Before the COVID-19 pandemic, infectious diseases accounted for only 1 of the top 10 leading causes of mortality in high-income countries, but 6 out of 10 in low-income countries [1]. The ongoing COVID-19 pandemic, which has already claimed more than 4.9 million lives at the time of publication, has shown that high-income countries are not immune to the impact of transmissible diseases. Unlike endemic diseases, which are often regionally confined by environmental factors, pandemics must be addressed with a comprehensive global plan to avoid the emergence of new, more dangerous variants and subsequent deaths. Thus, to effectively combat future pandemics, interventions must not only be developed rapidly but also made accessible to all people regardless of their level of income. 

There are a variety of approaches that can be integrated to improve comprehensive pandemic preparedness, including engineering resilient societal healthcare infrastructure, improving surveillance, leveraging systems vaccinology, further developing platform-based technologies, continuing our study of known pathogens that might be related to future pandemic pathogens, and performing early phase 0/I human clinical trials for promising vaccines [2,3,4,5,6,7]. The focus of this perspective is the development of “off-the-shelf” vaccine adjuvant platform technologies that can be rapidly deployed to mitigate mortality and morbidity during pandemics. One of the most effective means to address communicable diseases is to prevent people from contracting the disease and further spreading the pathogen. Vaccines play a critical role in ameliorating the global burden of infectious disease and remain one of our most important tools for preventing death by protecting people from symptomatic illness and reducing disease spread. Operation Warp Speed, a private-public partnership spearheaded by the United States government to accelerate COVID-19 vaccine development, has provided a blueprint for evaluating promising vaccine antigens on a short timeline by leveraging newly developed platforms such as mRNA [8]. Although vaccines are powerful tools in the fight against infectious diseases, it is extremely difficult to predict the next pathogen that will cause a pandemic before it begins. Further, there is no guarantee that the administration of antigens alone will be sufficiently immunogenic to confer prolonged protective immunity. In fact, this challenge has stymied the development of many vaccines for endemic diseases to date.

The earliest vaccines were killed whole-cell or live attenuated pathogens that were sufficiently immunogenic but generally caused more side effects than modern subunit and mRNA vaccines, which only use a small part of the pathogen, sacrificing some immunogenicity to improve safety [9]. Adjuvants improve vaccine efficacy by enhancing antigen immunogenicity, increasing the duration of the immune response, and/or controlling the type of immune response elicited. As a result, they can be used to improve immunogenicity in poorly responsive populations or enable antigen dose sparing [9]. Recent advances have also demonstrated that adjuvants can improve vaccine stability and thereby reduce reliance on cold-chain transportation, which remains a logistical challenge to distributing vaccines in low-resource settings [10,11]. Despite the importance of vaccine adjuvants, the translation of new adjuvants from the bench to the bedside has been incredibly slow. 

Since the discovery and regulatory approval of the first vaccine formulation containing an adjuvant in the 1920s, fewer than 10 adjuvants (Table 1) have been approved for use in humans [7]. Over the same time period, our understanding of the innate immune system, including its ability to detect pathogen-associated molecular patterns (PAMPs) through pattern recognition receptors (PRRs) on immune cells has drastically improved, and with it, so has our ability to engineer new, potentially more effective, adjuvants [7,12,13]. Some of the biggest challenges in executing rapid and comprehensive vaccination campaigns in response to pandemics are quickly producing a sufficient number of doses for global use, keeping cost low to ensure accessibility, and maintaining safety—all of which could potentially be addressed by adjuvants [14]. Thus, new antigen-agnostic adjuvants that leverage our growing ability to manipulate the immune system in a way that is both safe and confers robust immunity can help to improve preparedness for future pandemics. Advanced off-the-shelf adjuvants that have already been evaluated for safety and can be quickly tested with antigens against an emerging pathogen would allow researchers to improve the efficacy, development timeline, price, and supply of future vaccines.

## 2. Adjuvants in Clinically Approved Vaccines

When approving vaccines, the FDA considers adjuvants as part of the vaccine formulation. Therefore, adjuvants are not approved as a separate entity. The first adjuvant approved for human use as part of a vaccine was aluminum potassium sulfate, which was then replaced by aluminum hydroxide and aluminum phosphate, both known colloquially as alum. Although discovered a century ago, alum is still one of the most widely used adjuvants today. Alum has been shown to be effective with a variety of antigens, has been used in eight mainstream vaccines, and is currently being investigated for use in COVID-19 vaccines [7,15]. Alum primarily functions by causing inflammation and inducing cell death that leads to the release of endogenous danger-associated molecular patterns (DAMPs), such as extracellular DNA and ATP, which subsequently recruit immune cells to the injection site where vaccine antigens are present [7,16]. Despite the success of alum, vaccines formulated with this adjuvant have some negative side effects, such as injection site pain and redness, and fail to activate the CD8+ mediated cellular pathway of the adaptive immune system, which has been shown to improve vaccine efficacy for intracellular pathogens [17,18]. Additionally, there is evidence that the inclusion of alum may decrease the thermal stability of some vaccine antigens, which is of critical importance for global pandemic preparedness [19].

Alum remained the only adjuvant approved for use for most of the 20^th^ century until 1993 when virosomes were given regulatory approval. Virosomes and viral vectors can act as both delivery vehicles for protein and mRNA vaccines and adjuvants that stimulate the immune system. Virosomes are unilamellar liposomes composed of proteins and phospholipids from a virus (usually the same as the vaccine target). Virosomes were licensed for use in the flu vaccine, Inflexal, and the Hepatitis A vaccine, Epaxal, which have been administered more than 50 million times [20]. Virosomes function by leveraging the infective outer shell of the virus (devoid of any genetic material) to deliver antigen and cause inflammation through innate immune recognition that enhances both humoral and cellular immunity [21,22]. Viral vectored vaccines have been licensed for use in COVID-19 vaccines produced by Oxford/AstraZeneca and Johnson & Johnson, which use adenoviruses Ad5 and Ad26, respectively, to deliver mRNA coding for the SARS-CoV-2 spike protein antigen [22,23]. These vaccines differ from other COVID-19 mRNA vaccines from Pfizer/BioNTech and Moderna, which use non-adjuvanted lipid nanoparticles to deliver mRNA to cells [24]. Although the viral vector acts as an adjuvant by stimulating PRRs on innate immune cells, the body can also mount an adaptive immune response against the vector, which could lead to decreased efficacy with repeated doses due to the induction of carrier-neutralizing antibodies [25]. Antibodies against adenoviruses are also naturally occurring in the population due to environmental exposure. Epidemiological results have suggested that 77% of healthy adults have detectable levels of anti-Ad5 antibodies and 54% of the total adult population display high neutralizing antibody titers [26].

Initially used to help vaccinate the elderly against influenza by increasing the immunogenicity of the vaccine, MF59 is an oil-in-water emulsion that is approved in vaccines in 30 countries and has been administered to over 100 million people [7,27]. This adjuvant works by stimulating a MyD88-dependent pathway causing the secretion of chemokines and creating a local immunocompetent environment at the site of injection that enhances immune cell recruitment and antigen uptake [28]. The exact molecular mechanism of action remains unknown and is the subject of ongoing investigations [27]. Studies have shown that using the MF59 adjuvant in the H5N1 vaccine enabled dose sparing and increased cross-protection between different viral serotypes, which could help to mitigate concerns over vaccine production throughput, cost, and efficacy in a pandemic scenario [29]. AS03, another more recently approved oil-in-water emulsion, acts in a similar manner to MF59 and was shown to enhance cross-reactivity and dose sparing in H1N1 vaccines during the 2009 pandemic [30]. Unlike alum, which strongly favors humoral immune responses, MF59 and AS03 generate more balanced humoral and cellular immune responses [31,32].

The adjuvant effects of alum and oil-in-water emulsions arise from non-specific stimulation of the innate immune system. These systems were developed before there was a complete understanding of the innate immune system and the specificity of PRRs, and thus their serendipitous discovery as immune activators was not driven by knowledge of their mechanism of action. More recently developed adjuvants have used our new knowledge of PAMPs and PRRs to rationally design effective vaccine adjuvants. The most common PRR targets for these adjuvants are toll-like receptors (TLRs), which are membrane-bound receptors that recognize a variety of PAMPs and activate specific immune pathways that control effector response phenotype [33]. The proper stimulation of TLRs plays an important role in protecting the body from infection and insufficient activation can lead to ineffective antibody affinity maturation [34]. Among the most common TLR targets for vaccine adjuvants are TLR4 and TLR9, which sense bacterial endotoxin (LPS) and CpG (unmethylated cytosine phosphoguanosine dinucleotide) single-stranded DNA, respectively [7,35].

TLR4 is a cell surface transmembrane receptor that binds to Lipid A in LPS [35]. Although the ligand for TLR4 has been known since the late 1990s, LPS by itself is too cytotoxic to be an effective and safe vaccine adjuvant [36,37]. A detoxified form of lipid A, called MPLA (trademarked as MPL adjuvant^TM^ by GlaxoSmithKline), was developed and is a component in two adjuvants included in licensed vaccines: AS01 and AS04 [37,38]. AS01 is composed of liposomes that contain MPL and the immune activator and surfactant saponin QS-21, which has been shown to enhance both cellular and humoral immunity [39]. AS04, on the other hand, is composed of alum combined with MPL and is more efficacious than alum alone at improving the adaptive immune response [40]. Both adjuvants were designed to be platforms for use with a wide array of antigens and are present in multiple commercial vaccines. AS04 has been used in human papillomavirus and Hepatitis B vaccines and AS01 has been used in malaria and shingles vaccines [20]. 

TLR9, like TLR4, is a transmembrane protein but is located on the surface of the endosomal membrane rather than the cell membrane [9]. Although there are several CpG oligonucleotide sequences that have been studied as potential adjuvants, only CpG 1018 has been approved for use in humans [7,41]. CpG is a unique adjuvant because it elicits a relatively strong cellular immune response due to its interaction with TLR9, which, like the other TLRs found on the endosomal lumen, senses genetic material from intracellular pathogens [41]. Since intracellular pathogens are protected from antibodies while inside an infected cell, cytotoxic CD8+ T cells are necessary to kill the infected host cell and thereby prevent pathogenic replication [42]. In addition to generating robust cellular immunity, CpG 1018 has been shown to elicit a stronger humoral immune response in humans than alum in the case of the hepatitis B vaccine Heplisav-B [38].

## 3. Shortcomings of Current Adjuvants

Adjuvants in licensed vaccines provide an established and relatively well-understood platform to develop future vaccines since it is often easy to substitute a new antigen to change the disease target. Current adjuvants enable dose sparing, but the ideal pandemic vaccines would also be cost-effective and require as few doses as possible to mount a rapid and comprehensive vaccination campaign across the globe, including in low-resource settings. Currently, there are many pre-clinical and clinical trials investigating the use of these adjuvants with new vaccines, including those for COVID-19 [7,15]. Despite the successes of adjuvants currently included in licensed vaccines, GlaxoSmithKline’s malaria vaccine (RTS,S/AS01) and the SinoVac COVID-19 (CoronaVac) vaccine provide two case studies that demonstrate the shortcomings of current adjuvants. CoronaVac is formulated with alum and initially showed promising results [43]. Recent studies, however, have demonstrated that the vaccine provides less robust protection than the other commercially available COVID-19 vaccines, which is only marginally improved with additional doses, and is less effective in older populations [44,45]. More modern clinically approved adjuvants can also be insufficient in conferring robust immunity to infectious diseases. GlaxoSmithKline’s RTS,S/AS01 vaccine adjuvanted with AS01 protects only 36% of children after four doses delivered on an optimized, non-standard schedule [46]. This is an especially large burden relative to the vaccine’s value since the protection provided by the vaccine drops to effectively zero within seven years [47].

While additional doses of either RTS,S/AS01 or CoronaVac vaccine may improve their efficacy, given limited global vaccine supply and healthcare resources, this is logistically very challenging. Vaccine accessibility in resource-limited settings remains one of the largest barriers to mounting an effective pandemic response. Although there are several licensed COVID-19 vaccines, as of writing, only 1.9% of the population in low-income countries have received at least one dose compared to a global vaccination rate of 42.6% [48]. Wealthy nations should care about this because even if every individual in high-income countries were vaccinated, vaccine distribution to the rest of the world would still be critical to address from both a humanitarian perspective as well as means to effectively combat the COVID-19 pandemic in the long term. Unvaccinated populations serve as potential reservoirs for pathogen mutation, potentially giving rise to new variants with the ability to evade protection conferred with currently used vaccines. Another major barrier to vaccine distribution is antigen stability and the need for temperature-regulated storage [49]. Changes in vaccine formulation, including adjuvant selection, can help to stabilize antigens and eliminate the need for transportation within the cold chain [50]. Lastly, although there has been some success in eliciting cellular immunity with current adjuvants [51,52], most adjuvants that have been tested in humans fail to produce as strong of a cellular immune response as live attenuated viruses [9]. Thus, there is still a need to develop better adjuvants to elicit strong CD8+ mediated immunity necessary for fighting intracellular pathogens. Emerging adjuvants must address these issues to improve the viability of rapid global vaccination campaigns to address future pandemics.

## 4. Adjuvants with New Targets

There are many new adjuvants currently being investigated in both pre-clinical and clinical trials [7,20,22,53,54]. Many of these new technologies have novel targets in the innate immune system with the goal of boosting adaptive immunity and addressing the aforementioned issues with current adjuvants. Recent studies have demonstrated the potential of cell-penetrating peptides and antimicrobial peptides to adjuvant vaccines by improving antigen delivery and immunogenicity, respectively [55,56]. Probiotics are also being investigated as potential oral supplements to improve the immune response to COVID-19 vaccines [57]. The discovery of TLRs has initiated a revolution in adjuvant design. Although there are TLR9 and TLR4 agonists in current clinical vaccines, other TLR agonists are promising as well since they can leverage different immunological pathways. 

One of the most promising emerging targets is TLR5, which is a cell surface PRR that recognizes a highly conserved amino acid sequence on bacterial flagellin [58,59,60,61]. Stimulation of TLR5 leads to activation of dendritic cells and chemokine production, inducing a robust humoral response but a minimal cellular response [62,63]. This specificity for humoral immunity makes it an attractive adjuvant for vaccines targeting pathogens that can be neutralized by antibodies but not CD8+ T cells. Flagellin has been formulated as an antigen fusion protein and conjugated onto the surface of nanoparticles and virus-like particles with promising pre-clinical results [64,65,66]. Clinical trials using flagellin as an adjuvant for influenza vaccines in healthy adults (www.clinicaltrials.gov: NCT00921947, accessed on 23 October 2021) and people over the age of 65 (NCT00966238) have demonstrated its safety and efficacy. Flagellin has also been clinically tested in a pneumonic plague vaccine (NCT01381744) with similar success [67]. Although antibodies can be generated against flagellin, thereby decreasing its repeated efficacy, recent progress has been made in designing epitope-deficient flagellin that retains its adjuvant activity without generating anti-flagellin antibodies [68].

Other promising emerging targets include TLR7 and TLR8, which are structurally similar, located in the endosome, and recognize RNA and small molecules, such as imiquimod [38]. When stimulated with imiquimod or similar small molecule agonists, TLR7/8 induces both humoral and cellular immunity. Unfortunately, these molecules also exhibit poor water solubility and high toxicity [69]. However, formulation with delivery vehicles such as liposomes, nanoparticles, or nanofibers can enhance the safety, solubility, and immunogenicity of these adjuvants [70,71,72]. Several clinical trials have investigated TLR7/8 agonists as adjuvants in vaccines for influenza (NCT01737580, NCT03472976) and hepatitis B (NCT00175435, NCT03307902) among other diseases. Clinical trials investigating imiquimod as a topical adjuvant demonstrated its safety but had disappointing immunological results [73,74]. Ongoing clinical and preclinical trials are using new formulations of small molecule TLR7/8 agonists to improve their adjuvancy [75].

Although TLRs have been the molecular target of choice for most rationally designed adjuvants, there are other PRRs that are emerging targets for improving the immune response to co-delivered antigens, including C-type lectin receptors (CLRs), stimulator of interferon genes (STING), and others outside the scope of this perspective [42,76,77,78]. These different PRRs play important roles in a variety of innate immune pathways responsible for the effective response to a diversity of pathogens. 

STING is an intracellular PRR that senses cyclic dinucleotides (CDNs), which are a class of immunogenic bacterial and mammalian secondary messenger molecules. Several CDNs of both natural and synthetic origin have been investigated as potential adjuvants. Preclinical studies of vaccines adjuvanted with these CDNs have elicited cellular and humoral responses to an array of pathogens including influenza, tuberculosis, anthrax, *Streptococcus pneumoniae*, and methicillin-resistant *Staphylococcus aureus* [53]. CDNs have been shown to be more potent immunostimulatory compounds than CpG, alum, and LPS, and also elicit more balanced Th1/Th2/Th17 immune responses [79]. The long-lasting cellular and humoral protection generated by these adjuvants make them promising for use with vaccines targeting both intracellular and extracellular pathogens. As such, there are several ongoing clinical trials investigating CDNs as adjuvants for infectious disease and cancer immunotherapy (NCT02675439, NCT03010176, NCT03172936, and NCT03937141), which have, thus far, demonstrated broad safety in humans. Recent results from two CDN-adjuvanted cancer immunotherapy trials were disappointing, suggesting that further optimization of CDNs may be necessary to enhance their efficacy as adjuvants in infectious disease vaccines as well [53]. One barrier to CDN adjuvancy is that CDNs carry a net negative charge and, thus, inefficiently cross the negatively charged cell membrane, which is necessary to enter the cytosol and activate the STING pathway. To overcome this challenge, CDNs have been formulated with liposomes, oil-in-water emulsions, nanoparticles, and hydrogels to enhance cellular uptake and presentation to immune cells [80,81,82,83,84,85].

The innate immune system has evolved to recognize an array of pathogen-associated carbohydrates through CLRs on the surface of immune cells [77,86]. These receptors play an important role in infectious disease progression and protection, and using CLR agonists as adjuvants can help vaccines emulate natural infection [87,88,89,90]. As a result, there have been recent efforts investigating the use of several carbohydrate ligands of CLRs, including dextran, β-glucans, mannan, and chitosan, as vaccine adjuvants [91,92,93]. These carbohydrates have been formulated as nanoparticles, gels, and antigen-fusion complexes to improve both humoral and cellular immune responses to vaccines. These approaches have yielded promising pre-clinical results, including the induction of a balanced immune response and antigen dose sparing [94,95,96,97]. There are several ongoing and completed clinical trials investigating the safety and efficacy of carbohydrate adjuvants for infectious disease and cancer, such as chitosan (NCT00806962), β-glucan (NCT04936529), mannan [98], and crystalline δ-inulin under the trademark Advax^TM^ (NCT05005559) [94], which have thus far suggested carbohydrate adjuvants can be well-tolerated and effective in humans at the doses necessary to confer an immunological benefit [98,99,100]. Carbohydrate PAMPs are also recognized by PRRs other than CLRs [54,94,100]. For instance, the previously mentioned saponin QS-21 adjuvant is already licensed for use as part of AS01, but is not known to interact with any CLRs [94].

## 5. Emerging Adjuvant Platforms

Due to the great clinical interest in adjuvants that can be applied to a variety of current and future pathogens, many groups have been working on developing the next generation of antigen-agnostic adjuvant platforms. This section highlights several systems that show substantial promise for improving pandemic preparedness.

**Figure 1:**
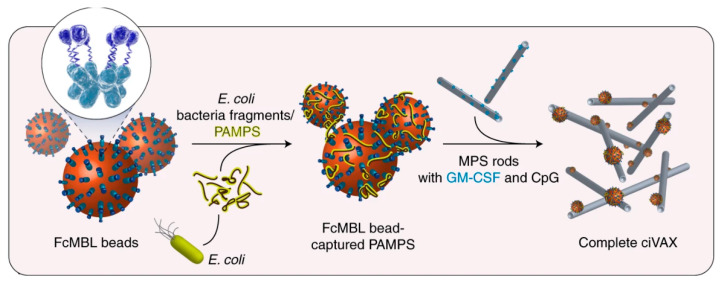
Formulation of ciVAX from E. coli lysate. Adapted and reprinted by permission from Springer Nature, *Nature Biomedical Engineering* [104]. Copyright 2021.

The Mooney Group at Harvard University has been developing self-assembling mesoporous silica rods (MPS) modified with granulocyte-macrophage colony-stimulating factor (GM-CSF) and CpG as a flexible vaccine adjuvant platform for numerous antigens [101,102]. Single injections of MPS adjuvant generated strong humoral responses to substances such as nicotine and cancer neoantigens that persist for at least six months after vaccination. These vaccines generated higher antibody titers and memory B cell responses than antigen alone or antigen adjuvanted with alum. MPS persistence at the injection site and immune cell recruitment to the injection site were shown to be critical to the efficacy of the adjuvant [103]. Most recently, the group has developed a modular platform for bacterial vaccines using engineered opsonin Fc-mannose-binding lectin- (FcMBL) coated magnetic particles. FcMBL binds to carbohydrate-containing PAMPs and antigens from lysed bacteria, which are then mixed with MPS, GM-CSF, and CpG to construct ciVAX (Figure 1). Thus, a new vaccine can be rapidly generated against a bacterial pathogen by switching to a new, relevant bacteria cell lysate that is mixed with the FcMBL beads. The efficacy and safety of ciVAX were demonstrated in mice with lysate from methicillin-resistant *Staphylococcus aureus* and in pigs with *E. coli* lysate. In both cases, ciVAX prophylaxis protected animals from bacterial challenge by generating a balanced humoral and cellular immune response. It was also demonstrated that all the components of ciVAX could be applied to other scaffolds for similar effects, thus opening the opportunity for combining this approach with other emerging biomaterial-based strategies [104]. This approach may be further expanded to other pathogens by studying the vaccine efficacy of ciVAX formulated with PAMPs and antigens from killed parasites or viruses.

Self-assembling peptides are promising biomaterial adjuvants due to their inherent biocompatibility, tunability through altering their amino acid sequence, and scalability using solid-phase peptide synthesis [105]. The Collier Group at Duke University has developed a self-adjuvating fiber-forming peptide Q11 (Ac-QQKFQFQFEQQ-Am) that generates a balanced immune response when conjugated to a variety of antigens. The efficacy of this system was first demonstrated in mice using the model ovalbumin peptide antigen OVA_323-339_ and has since been used with model folded protein antigens such as green fluorescent protein (GFP) and clinically relevant peptide antigens from pathogens including human immunodeficiency virus (HIV), influenza, *Mycobacterium tuberculosis*, and *Staphylococcus aureus* [106,107,108,109,110,111]. Studies with ovalbumin epitopes have demonstrated that high-affinity B cell responses can be elicited in the absence of inflammation, enabling the platform to be effective with a low risk of toxicity or side effects [112]. The immune response to this platform can be easily tailored between humoral and cellular immunity by changing the ratio of B cell and T cell epitopes displayed on the self-assembling peptide, which allows for tunability for future vaccines [107]. This platform has been shown to be effective when administered sublingually and intranasally, potentially reducing the need for administration by trained medical professionals in low-resource settings [111,113]. Self-assembled peptide vaccine adjuvants have been shown to improve vaccine stability, which is of high interest for improving pandemic preparedness [114]. The Collier Group has improved upon this feature of self-assembling peptide adjuvants in a recent study where they formulate Q11 as a thermostable solid tablet for sublingual administration. They found that this formulation induced high levels of circulating antigen-specific IgG, which could potentially reduce reliance on the cold chain and reduce the need for biohazardous disposal of used syringes, thereby lowering cost and improving accessibility [110].

During the COVID-19 pandemic, mRNA technology has been a key in the rapid development of vaccines and is likely to play an important role in addressing future pandemics as well—possibly with accelerated development and scale-up due to the buildup of supportive infrastructure and newfound precedence in clinical use. Thus, new adjuvants that synergize with mRNA vaccines may be very important in increasing vaccine efficacy, affordability, and stability. Currently, lipid-based delivery systems are used in mRNA vaccines because they demonstrate high transfection efficiency; however, they also require specialized storage to remain stable and lack inherent adjuvant activity [115,116]. New nanoparticle adjuvants show promise in improving mRNA vaccine safety, efficacy, stability, and cost [117,118]. Several groups have been developing polymer adjuvant formulations using modified polyethyleneimine (PEI) and polylactic acid (PLA) particles to deliver antigen and adjuvant to antigen-presenting cells. These are promising materials due to their modularity, ease of manufacturing, stability, and status as materials with precedence in FDA-approved formulations. Although these materials have lower transfection efficacies and worse toxicity profiles than liposomes, recent advances have shown that these issues can be addressed by formulation modifications [117]. Altering polymer molecular weight, charge, and surface modification can all impact mRNA transfection and adjuvancy, thereby offering the potential to tailor the immune response as desired [117]. These particles have been shown efficacious for antigens from pathogens such as HIV-1, rabies, and influenza, and have been shown to be well-tolerated and effective in humans when included in a vaccine (NCT02241135) [119,120,121]. The adjuvant activity and control of the immune effector response of these materials can be improved by loading them with PRR agonists [122,123,124]. Polymer nanoparticle formulations have been found to target and persist in lymph nodes for effective mRNA delivery to the immune system [124]. PEI and PLA mRNA vaccines have been administered through a variety of different routes, opening the opportunity for cost reduction and enhancing the feasibility of rapid deployment during future pandemics [117].

## 6. Discussion: Perspectives for Adjuvant Translation to Pandemic Preparedness

Although we cannot predict precisely what pathogen will cause the next pandemic, we can rationally engineer and characterize adjuvants to promote specific immune responses across a broad array of antigens so that when a new pathogen and immunity-conferring antigen are identified, we can rapidly develop a potent vaccine. This might be especially important if we do not have the benefit of previous knowledge of immunity-conferring vaccine antigens like we had this time for COVID-19 due to existing research into the related severe acute respiratory syndrome coronavirus (SARS-CoV-1). 

One potentially valuable approach to preparing for the next pandemic would be to create a library of well-characterized, “ready-to-use” adjuvant platforms. Ideally, these adjuvants would have previously demonstrated the ability to promote a robust and protective immune response for a diverse set of antigens so that pandemic antigens can be rapidly tested with promising candidates to determine the most effective vaccine formulation. Although there are many adjuvants currently in preclinical development that could improve global readiness for future pandemics, the sluggish adoption of new adjuvants can be attributed to safety concerns, regulatory hurdles, and issues with technology transfer. Safety is one of the biggest public concerns when it comes to the development of new vaccines and vaccine adjuvants, and, due to the complexity of the immune system, an adjuvant’s safety with one antigen does not necessarily prove its safety with all antigens. Consequently, adjuvants will ultimately be approved for use only as part of a specific vaccine formulation rather than receiving blanket approval. Unfortunately, this reduces the financial incentives for vetting adjuvant safety without a specific vaccine in mind. Additionally, without extensive preceding safety studies, companies developing vaccines for emerging pandemics are disincentivized from testing their new antigen with a “risky” adjuvant, even if it has the potential for superior immunological outcomes [9]. To address these issues, prospective public- or foundation-funded high-throughput safety testing of emerging adjuvants could be developed to establish a broad safety profile with a variety of antigens and thereby de-risk the incorporation of adjuvants in vaccines developed in response to a pandemic. Non-human primate (NHP) safety studies are the best way short of human testing to demonstrate adjuvant safety but require many animals, which raises both financial and ethical concerns during vaccine development [125].

**Figure 2:**
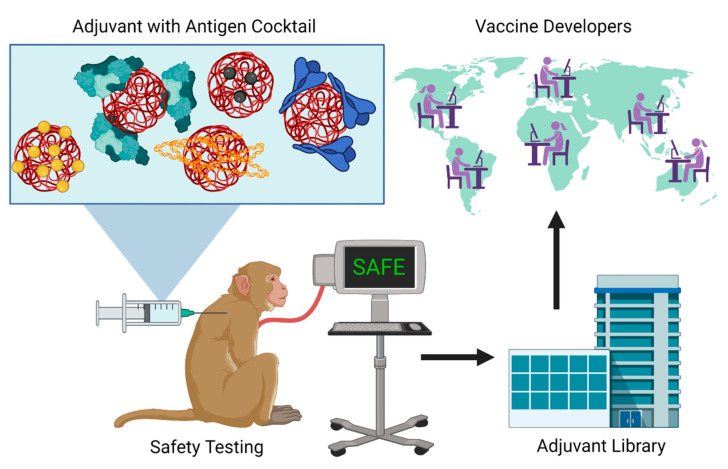
A single adjuvant can be mixed with multiple antigens for safety testing in NHP. Standardized adjuvant formulations and protocols can then be shared with the adjuvant library for global distribution to vaccine developers for rapid vaccine development to pandemic antigens.

One strategy that could be used to pre-qualify adjuvant safety in NHPs in a more cost-effective manner would be to test them in combination with an antigen cocktail. Instead of testing the safety of adjuvants pairwise with one antigen at a time, which would be prohibitively expensive, these experiments could test formulations with one adjuvant and multiple vaccine antigens (Figure 2). For example, instead of testing 10 antigens from different pathogens with an adjuvant in five primates each (total of 50 animals), all 10 antigens can be mixed and tested with the adjuvant at once to significantly reduce cost and the number of animals needed. Although the antigen cocktail would not necessarily be useful in determining vaccine efficacy to individual antigens and may not be fully representative of real-world vaccines, this approach could provide a higher throughput means to establish safety profiles of adjuvants and de-risk future experiments for their clinical use while using fewer animals. Once an adjuvant is demonstrated to be well-tolerated using this initial screen, further investigation into its efficacy with emerging pandemic antigens can be explored with the knowledge that the adjuvant will likely be well-tolerated in humans. 

Another barrier to adjuvant adoption in new vaccines is technology transfer, as the groups developing new adjuvants are often not the same groups developing new antigens for emerging diseases [126]. Thus, once adjuvants have passed the “antigen cocktail” safety screen, researchers developing adjuvants could be encouraged to provide materials and protocols with a centralized public antigen library modeled similarly to the National Institutes of Health (NIH) tetramer-core operated by Emory University (Figure 2). Researchers and companies would also be financially incentivized to share their technology as it increases the likelihood that it will be translated to the clinic and produce licensing revenue. This library would allow researchers to test new antigens on a plethora of off-the-shelf standardized adjuvant platforms produced using uniform protocols and verified to be endotoxin-free to allow for the easy comparison of data between experiments and research groups. A core like this may also succeed in facilitating more collaborations between groups that develop antigens and adjuvants to streamline vaccine production. In a pandemic setting, access to as many resources as possible is critical to rapidly find the best adjuvant, and this library will connect antigen and adjuvant developers so they can work together in finding the next pandemic-stopping vaccine.

Although more work is necessary before the global community is ready to confront the next pandemic, lessons learned from past and current pandemics can be used to guide future adjuvant development and testing. Calls for reform and new platform development have been sounded in the past and have been eerily accurate in predicting our lack of pandemic preparedness [6]. Now, equipped with experience from the COVID-19 pandemic and new funding opportunities that target pandemic preparedness such as the ARPA-H initiative from the NIH (https://www.nih.gov/arpa-h, accessed on 23 October 2021), there is new hope that the next novel pathogen will be rapidly met with adjuvanted vaccines that are safe, effective, and accessible to enable a comprehensive global response.

## Figures and Tables

**Table 1:** List of adjuvants in vaccines approved for human use with their composition and known mechanism of action.

Adjuvant (Year)	Composition	Licensed Vaccine Targets	Immunological Function
Alum (1926)	Suspension of aluminum hydroxide or aluminum phosphate salts	Anthrax, hepatitis A, hepatitis B, human papillomavirus, diphtheria-pertussis-tetanus (DPT and TdaP), haemophilus influenzae type b, Japanese encephalitis, pneumococcal conjugate vaccines, and COVID-19	Releases DAMPs at injection site by causing cell death, resulting in the recruitment and activation of dendritic cells and neutrophils
Virosomes (1993)	Unilamellar liposomes composed of viral proteins and phospholipids of vaccine target virus	Seasonal flu and hepatitis A	PAMPs on the surface of virosomes stimulate and activate antigen-presenting cells while also facilitating antigen delivery
MF59 (1997)	Emulsion of Squalene, Tween (polysorbate) 80, and Span 85	Seasonal flu and pandemic flu (H1N1)	Known to recruit and activate macrophages and dendritic cells and cause chemokine secretion
AS03 (2009)	Emulsion of Squalene, α-tocopherol, and Tween (polysorbate) 80	H1N1	Activates human monocytes and macrophages and induces NF-κB activity and chemokine production
AS04 (2009)	MPL adsorbed onto alum	Human papillomavirus and hepatitis B	MPL activates TLR4 and NF-κB to stimulate antigen presenting cells and innate immune system while alum causes the release of DAMPs and local inflammation
AS01 (2015)	Liposome co-delivery of saponin QS-21 and MPL	Malaria and herpes zoster	Activates TLR4 in innate immune cells and caspase 1 in subcapsular sinus macrophages, induces differentiation of monocytes to DC, and activates NF-κB and production of IFNγ
CpG 1018 (2018)	22 nucleotide single-stranded DNA containing unmethylated cytosine phospho-guanosine dinucleotide	Hepatitis B	Activates TLR9 resulting in a type I interferon response
Viral Vectors (2020)	Adenoviruses carrying mRNA encoding for protein antigen	COVID-19	PAMPs on the surface of adenoviral carriers activate the innate immune system while also facilitating transfection for mRNA vaccines

## Data Availability

Not applicable.
